# Downregulation of lncRNA-PVT1 participates in the development of progressive chronic kidney disease among patients with congestive heart failure

**DOI:** 10.1186/s12950-021-00293-5

**Published:** 2021-09-25

**Authors:** Yingwei Chang, Chunmei Liu, Jing Wang, Jing Feng, Yulan Chen, Mufang Qi, Yaguang Guo

**Affiliations:** 1 Department of Geriatrics, Qingdao Huangdao district central hospital, Qingdao City, Shandong Province 266000 PR China; 2 Hemopurification Center, The Second Affiliated Hospital of Medical College of Qingdao University, Qingdao, 266000 Shandong Province PR China; 3 Department of Nephrology, The Second Affiliated Hospital of Medical College of Qingdao University, Qingdao, 266000 Shandong Province PR China; 4 Department of Nephrology, Affiliated Hospital of Shaoxing College of Arts and Sciences, No.999 South Central Road, Shaoxing, 312000 Zhejiang Province PR China

**Keywords:** Congestive heart failure, Chronic kidney disease, PVT1, Diagnosis, Prediction

## Abstract

**Background:**

Congestive heart failure (CHF) is a major cause of the development of progressive chronic kidney disease (CKD), while the mechanism is still unknown. LncRNA PVT1 contributes to kidney injury. This study aimed to explore the role of PVT1 in the development of CKD in CHF patients.

**Methods:**

Expression of PVT1 in plasma samples of CHF patients with and without CKD was determined by RT-qPCR. The diagnostic value of plasma PVT1 for CKD was evaluated by ROC curve analysis. The predictive value of PVT1 for the development of CKD in CHF patients was analyzed by a 2-year follow-up study. Changes in PVT1 expression in CKD patients during treatment were analyzed by RT-qPCR and reflected by heatmaps.

**Results:**

Plasma PVT1 was downregulated in CHF and further downregulated in CHF patients complicated with progressive CKD. ROC curve analysis showed that plasma PVT1 levels could be used to distinguish CHF patients complicated with CKD from CHF patients without CKD and healthy controls. During a 2-year follow-up, patients with high CHF levels had a low incidence of progressive CKD among CHF patients. Moreover, with the treatment of progressive CKD, plasma PVT1 was upregulated.

**Conclusions:**

LncRNA-PVT1 downregulation may participate in the development of progressive CKD among patients with CHF.

## Introduction

Congestive heart failure (CHF), also known as heart failure, is a common clinical disorder reflected by reduced heart function and/or impaired heart structure [1]. It is estimated that more than 6.5 million people in the US are suffering from CHF, resulting in more than 30 billons of medical cost [2]. Patients with CHF usually show dyspnea and fatigue at rest. Besides that, CHF is also correlated with a high mortality rate even with active treatment. About 10% of CHF patients will die of CHF within 1 month after treatment, and the 5-year overall survival rate is only about 50% [3, 4]. In CHF patients, elevated central venous pressure may be transmitted to a glomerular efferent arteriole, leading to reduced glomerular filtration rate (GFR) and glomerular filtration pressure, which may cause the development of chronic kidney disease (CKD) [5, 6]. Therefore, the development of CDK contributes to the high mortality of CHF.

CDK prevention and effective treatment in CHF patients are critical for improving the survival of CHF patients [5, 6]. However, the molecular mechanisms linking CHF and CKD are unclear, limiting the development of preventative and treatment approaches [7]. Without protein-coding capacity, lncRNAs participate in CHF and CKD mainly by regulating related gene expression [8–10]. Compared to protein-coding genes, lncRNAs are more spatially and temporally expressed with more specific functions [11]. Therefore, lncRNAs may be promising targets to understand the mechanism that mediates CKD development in CHF. However, the function of most lncRNAs in CDK and CHF remains unclear. A recent study showed that lncRNA PVT1 participates in LPS-induced septic acute kidney injury [12]. In addition, our preliminary microarray analyses revealed altered PVT1 expression in both CHF patients with and without CKD. Therefore, our study was performed to explore the potential involvement of PVT1 in the development of CKD in CHF.

## Materials and methods

### Patients and healthy controls

From June 2016 to June 2018, a total of 50 healthy controls (28 males and 22 females), 100 CHF patients with obvious complications (CHF group, 56 males and 44 females), and 50 CHF patients complicated with CKD (CHF + CKD group, 28 males and 22 females) at Affiliated Hospital of Shaoxing College of Arts and Sciences were included in the study. They were at the age of 46 to 68 years with a median of 57 years. All healthy controls showed normal physiological functions in systemic physiological exams. CKD was diagnosed based on urine and blood tests. CHF was diagnosed based on the fluid in the lungs on chest X-ray, heart size and/or blood flow to the heart muscle on electrocardiogram, heart rate, heart rhythm, and ventricle size. All participants signed informed consent. The study was approved by the Ethics Committee of our hospital.

### Treatment and plasma preparations

The 50 patients in the CHF + CKD group were treated by optimized dialysis, angiotensin-converting enzyme inhibitors, or fluid overload control according to patients’ disease conditions and health conditions. Prior to therapy, fasting blood (2 ml) was extracted from all three groups of patients. During treatment, fasting blood was also extracted from CHF + CKD group at 1, 2, and 3 months after the treatment. All blood samples were mixed with citrate at a ratio of 1:10 in centrifugation tubes, followed by centrifugation at room temperature for 20 min at 1200 g. The supernatant (plasma) was collected and kept in liquid nitrogen.

### Follow-up (2-year) of CHF group

To explore the predictive value of plasma PVT1 for CKD development in CHF patients, the 100 CHF patients were followed up for 2 years. Patients who died during the follow-up before the diagnosis of CKD were excluded. Follow-up was performed in a monthly manner through the outpatient visit.

### RNA preparations

Total RNAs were extracted from all plasma samples using RNAzol (Sigma-Aldrich) and treated with DNase I (Invitrogen) for 2 h at 37 °C to remove genomic DNAs. RNA integrity was analyzed on a 5% urea-PAGE gel and Agilent 2100 Bioanalyzer. RNA purity was analyzed by OD26/280 ratio. Samples with RNA integrity value (RIN) higher than 9 and OD260/280 ratio around 2.0 were used in the subsequent experiments.

### RT-qPCR assay

RNA samples with satisfactory quality were reverse transcribed into cDNA using SS-RT-IV kits (Invitrogen). To determine PVT1 expression, qPCRs were performed using SYBR Green Master Mix (Bio-Rad) with 18S rRNA as the internal control at conditions of denaturation at 95 °C for 1 min followed by 40 cycles of 10s at 95 °C and 55 s at 58 °C. Three technical replicates were included in each experiment. The relative Ct values of PVT1 were calculated as ΔCt = Ct (PVT1) – Ct (18S rRNA). The sample with the biggest ΔCt value was set to value “1”, and all other samples were normalized to this sample. Primer sequences used in PCR were PVT1 forward 5′-TGAGAACTGTCCTT ACGTGACC-3′ and reverse 5′-AGAGCACCAAGACTGGCTCT-3′ and 18S rRNA forward 5′-CTACCACATCCAAGGAAGC-3′ and reverse 5′-TTTTCGTCACTACCT CCCCG-3′.

### Cardiomyocytes and treatment

Human AC16 cardiomyocytes (EMD Millipore, USA) were cultured in DMEM supplemented with 1% penicillin, 1% streptomycin, and 12% FBS at 37 °C in an incubator with 5% CO_2_. Cells were treated with 0, 1, 1, 2, and 5 μg/ ml LPS for 24 h prior to analyzing PVT1 expression.

### Statistical analysis

PTV1 levels in plasma samples were expressed as mean values of three technical replicates. PVT1 expression levels during the treatment were represented by heatmaps plotted using Heml 1.0 software. The 100 CHF patients were divided into high and low PVT1 level groups (*n* = 50; cutoff value = the median PVT1 level in plasma samples of CHF patients). CKD-free curves were plotted and compared by log-rank test. *P* < 0.05 was considered statistically significant.

## Results

### Plasma PVT1 was downregulated in CHF and further downregulated in CHF + CKD

**Fig. 1:**
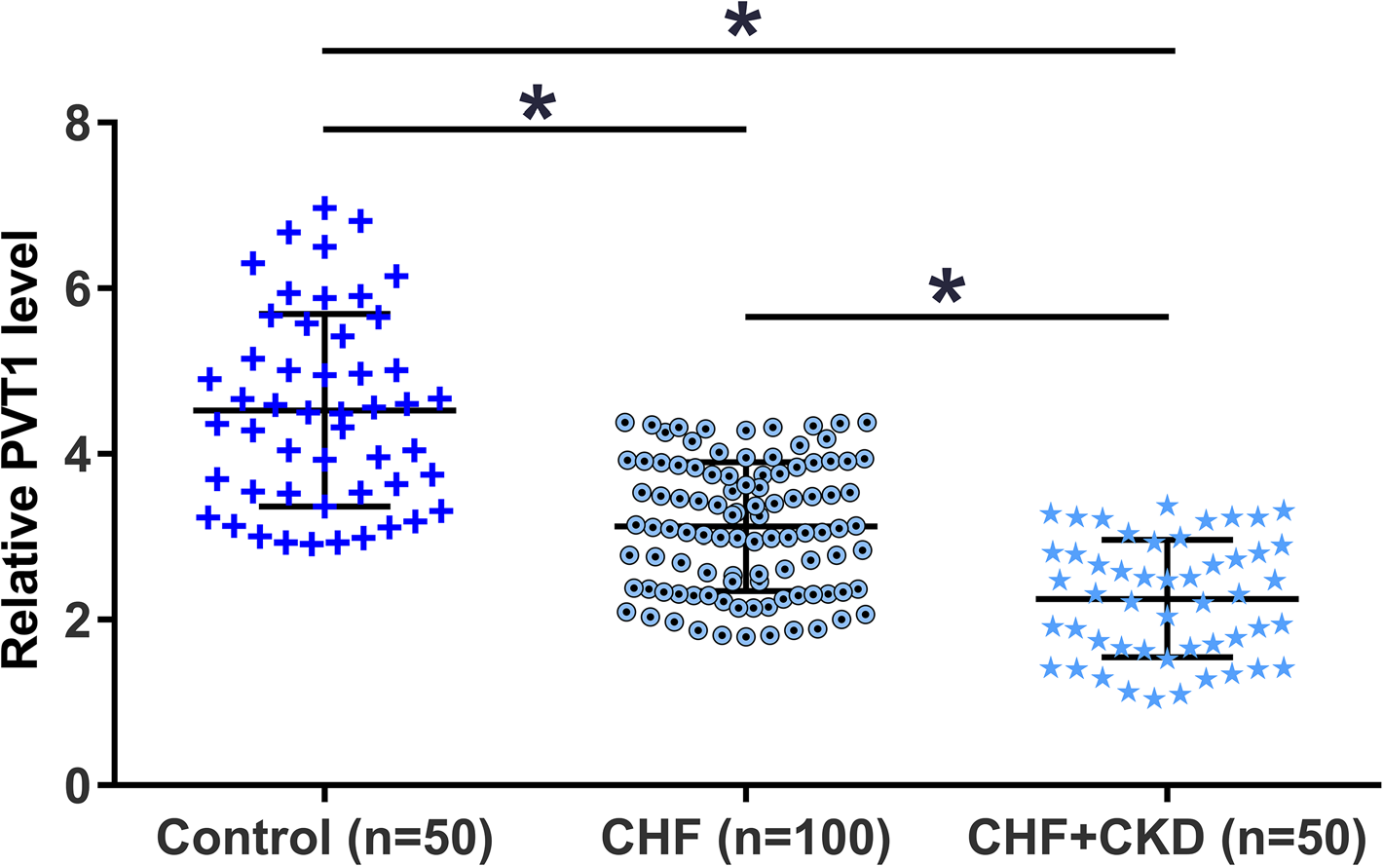
Plasma PVT1 was downregulated in CHF and further downregulated in CHF + CKD. PVT1 levels in plasma samples collected from the control group (*n* = 50), CHF group (*n* = 100), and CHF + CKD group (*n* = 50) were measured by RT-qPCR. PTV1 expression levels in plasma samples from the three groups of patients were expressed as the mean values of three technical replicates. Data were compared by unpaired t test. ** *p* < 0.01

PVT1 levels in plasma samples collected from the control group (*n* = 50), CHF group (*n* = 100), and CHF + CKD group (*n* = 50) were measured by RT-qPCR. Plasma PVT1 levels were significantly lower in CHF (1.65-fold) and CHF + CKD (2.22-fold) groups than in the control group (*p* < 0.01). Moreover, PVT1 levels were also significantly lower in the CHF + CKD group (1.33-fold) than in the CHF group (Fig. 1, *p* < 0.01). Therefore, altered PVT1 expression may participate in CHF and CKD.

### Altered plasma PVT1 levels separated CHF + CKD patients from CHF patients and healthy controls

**Fig. 2:**
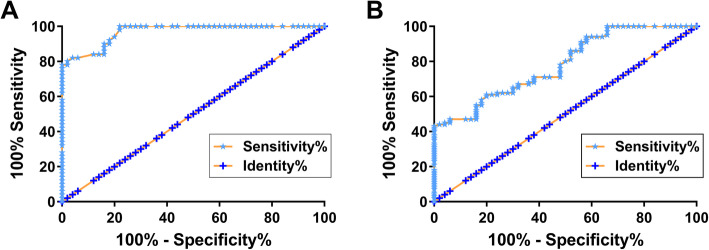
Altered PVT1 plasma levels separated CHF + CKD patients from CHF patients and healthy controls. ROC curve analysis was performed to analyze the diagnostic value of plasma PVT1 for CKD. In ROC curve analysis, patients with CHF + CKD were considered as the true positive cases, and healthy controls (A) or CHF patients (B) were considered as the true negative cases

ROC curve analysis was performed to analyze the diagnostic value of plasma PVT1 for CKD. The area under the curve (AUC) was 0.9744 when patients with CHF + CKD and and the controls were considered the true positive cases and the true negative cases, respectively, with a 95% confidence interval of 0.9512 to 0.9976 and standard error of 0.01181 (Fig. 2A, *p* < 0.0001). When CHF patients were considered the true negative cases, AUC was 0.7821 with a 95% confidence interval of 0.7084 to 0.8558 and standard error of 0.03758 (Fig. 2B, *p* < 0.0001). Therefore, altered PVT1 expression in plasma may assist the diagnosis of CKD in CHF patients.

**Fig. 3:**
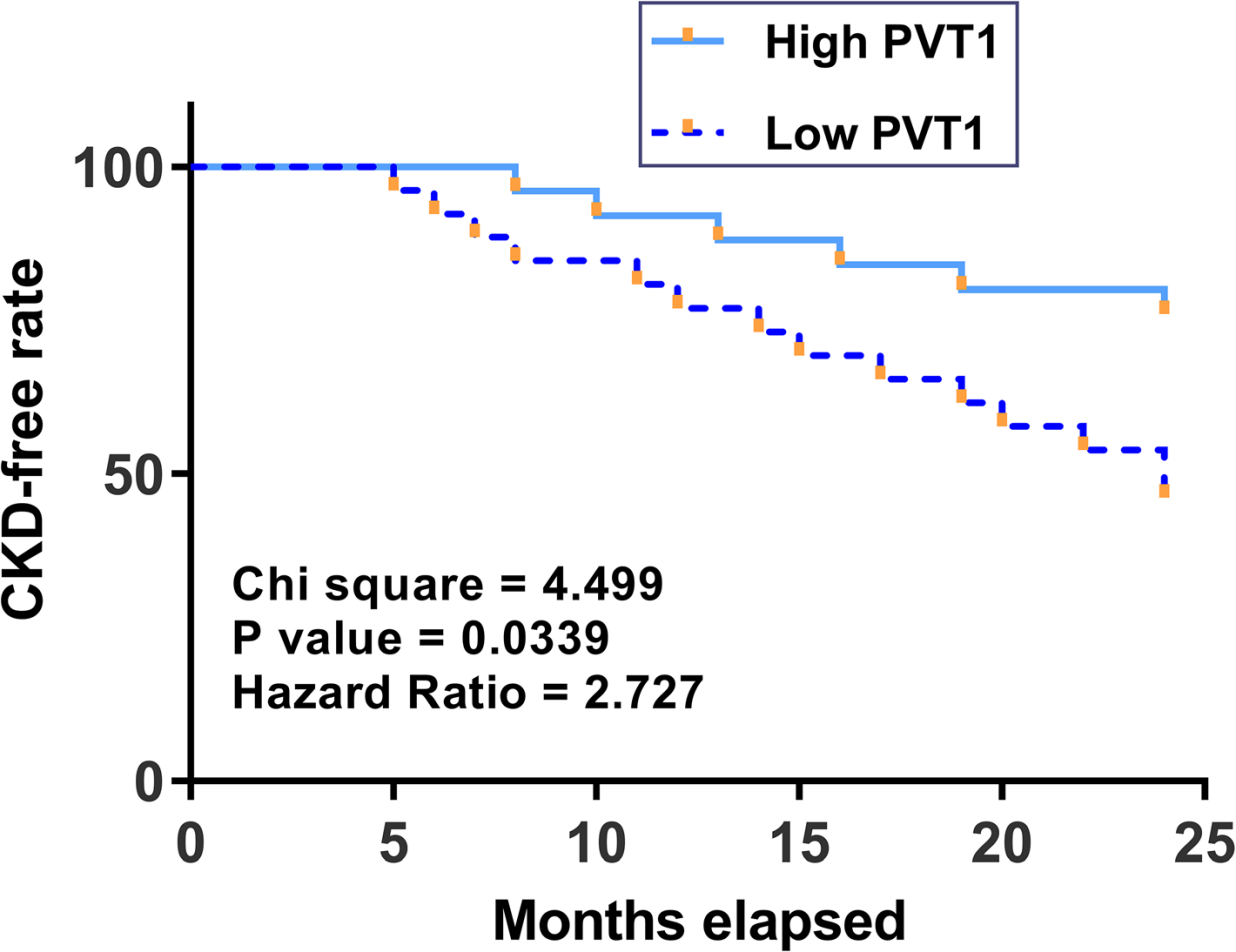
Follow-up analysis of CHF group revealed the predictive value of plasma PVT1 for CKD in CHF patients. The 100 CHF patients were divided into high and low PVT1 level groups (*n* = 50; cutoff value = the median level of PVT1 in plasma samples of CHF patients). CKD-free curves were plotted and compared by log-rank test

### Predictive value of plasma PVT1 for CKD in CHF patients

The 100 CHF patients were followed up for 2 years to analyze the predictive value of plasma PVT1 for CKD. CKD-free curves were plotted for both high and low PVT1 level groups and compared using the log-rank test (Fig.3). Compared to the high PVT1 level group, the low PVT1 level group showed significantly higher incidence rate of CKD.

### Plasma PVT1 levels increased in the CHF + CKD group during the follow-up

**Fig. 4:**
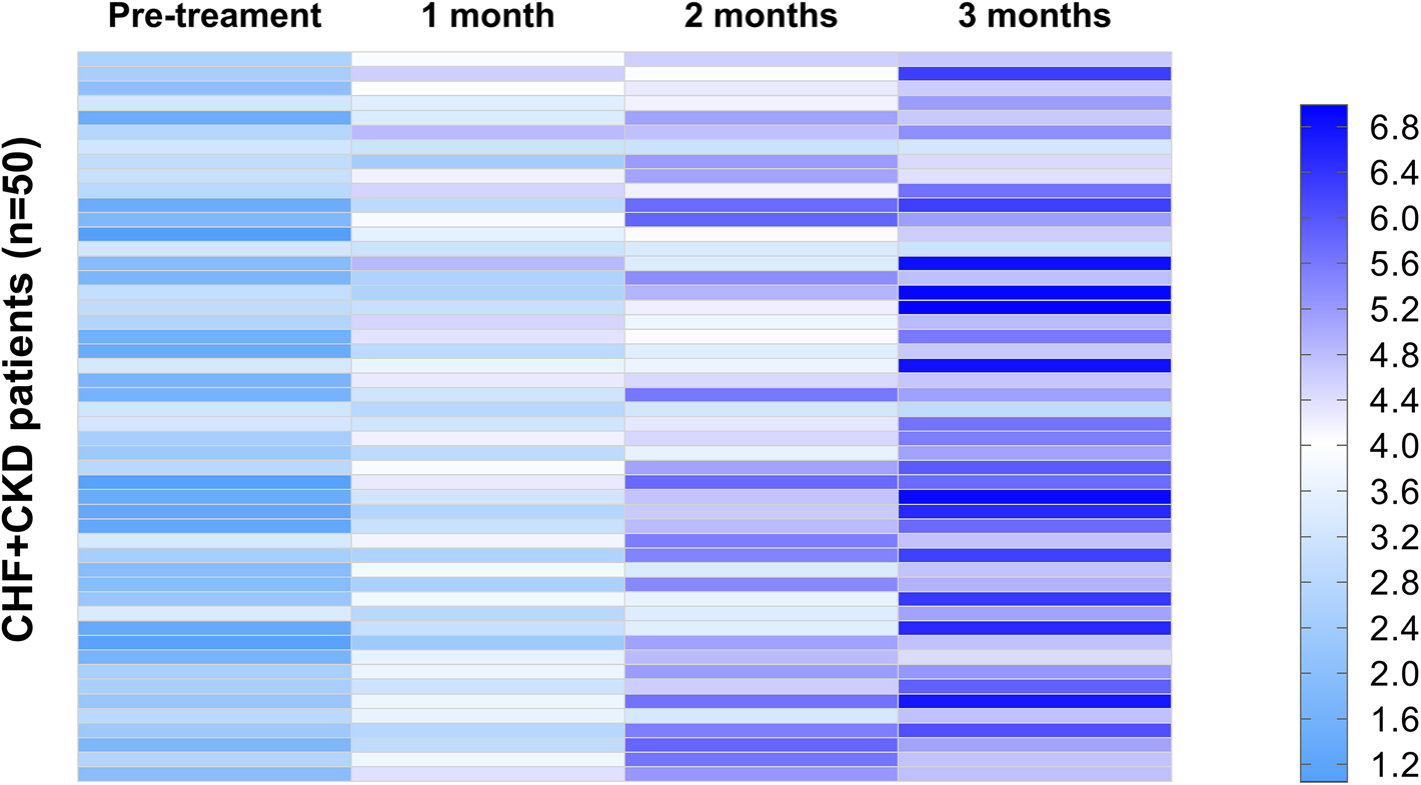
Plasma PVT1 levels increased in CHF + CKD group during the follow-up. Plasma PVT1 levels in CHF + CKD group were measured prior to the treatment, and at 1, 2, and 3 months after the beginning of the treatment. Heatmaps were plotted using Heml 1.0 software to reflect the changes in plasma PVT1 levels

Plasma PVT1 levels in the CHF + CKD group were measured prior to the treatment and at 1, 2, and 3 months after the beginning of the treatment. Heatmaps were plotted using Heml 1.0 software to reflect the changes in plasma PVT1 levels. It was observed that plasma PVT1 levels increased during the treatment (Fig. 4). Compared to the pre-treatment PVT1 levels, 1.8-, 2.2-, and 2.9-fold increases were observed at 1, 2, and 3 months after the beginning of the treatment, respectively.

### LPS treatment decreased PVT1 expression in cardiomyocytes

**Fig. 5:**
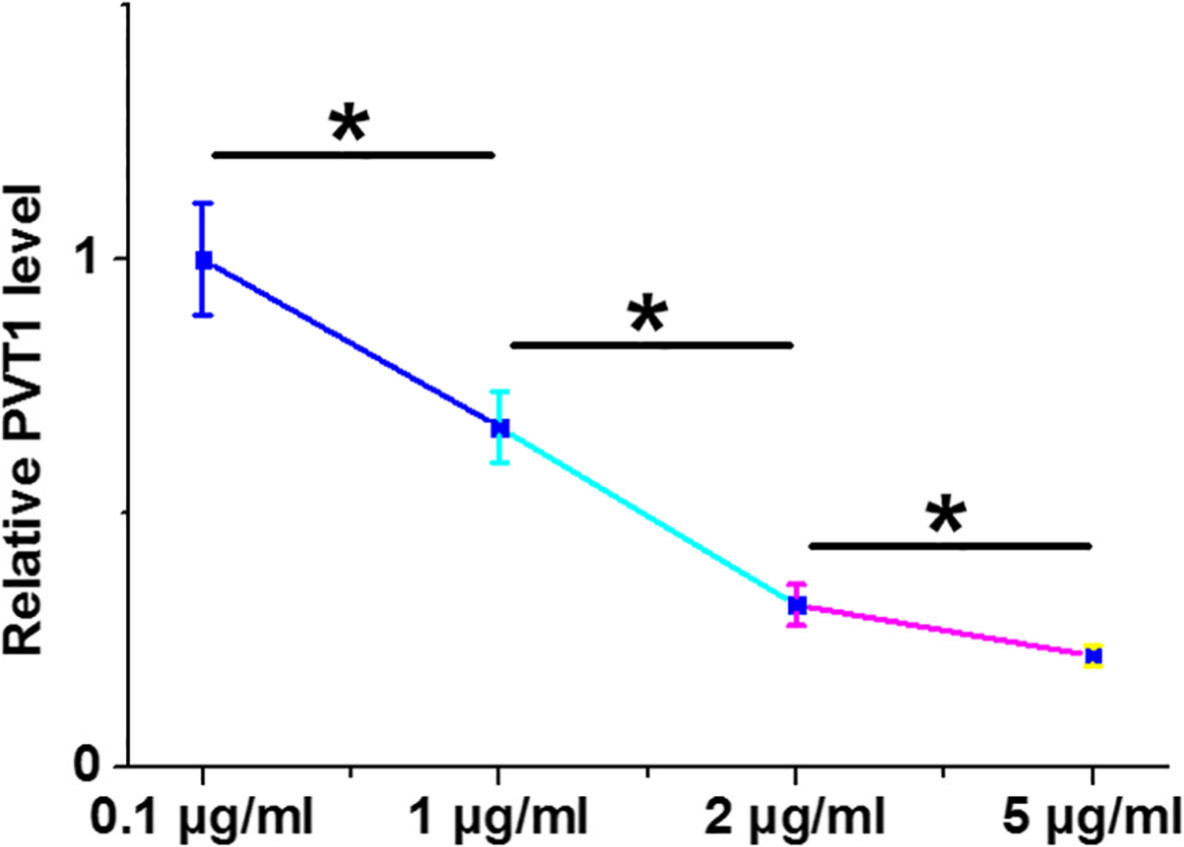
LPS treatment decreased PVT1 expression in cardiomyocytes. AC16 cells were treated with 0.1, 1, 2, and 5 μg/ml LPS for 24 h, followed by RNA isolations and RT-qPCRs to determine the expression of PVT1. * *p* < 0.05

AC16 cells were treated with 0.1, 1, 2, and 5 μg/ml LPS for 24 h, and PVT1 expression levels were determined using RT-qPCRs after RNA isolations. It was observed that LPS treatment decreased PVT1 expression in a dose-dependent manner (Fig. 5, *p* < 0.05).

## Discussion

This study mainly explored the differential expression of PVT1 in CHF and CKD and analyzed the diagnostic value of PVT1 for the development of CKD in patients with CHF. We found that PVT1 was downregulated in both CHF and CKD, and altered PTV1 expression may have diagnostic and prognostic values for CKD.

It has been reported that PVT1 is upregulated in LPS-induced septic acute kidney in mice model and promotes disease progression by regulating JNK/NF-κB and TNFα pathways [13]. Curcumin could protect LPS-induced septic acute kidney injury by suppressing PVT1 expression [12]. Moreover, PVT1 knockdown targets miR124 to improve vancomycin-induced acute kidney injury through the activation of NF-κB signaling [14]. Therefore, PVT1 can promote the development of LPS- and vancomycin-induced kidney injury. Interestingly, our study showed that PVT1 was downregulated in CHF and CKD, suggesting the different role of PVT1 in CHF-induced CKD. However, the functions of PVT1 in CHF-induced CKD remain to be further explored.

The development of CKD is a major cause of death in CHF patients [5, 6]. Therefore, it is important to accurately diagnose and predict CKD among CHF patients. In our study, we showed that altered plasma PVT1 expression separated CHF + CKD patients from both CHF patients and healthy controls. Moreover, during the follow-up study, low PVT1 levels were found to be closely correlated with a high incidence rate of CKD among CHF patients. Therefore, altered PVT1 expression in CHF patients may predict the occurrence of CKD.

During the treatment, we observed continuous PVT1 increased plasma levels in CHF + CKD patients. The increased PVT1 expression may promote the recovery of patients. In addition, altered PVT1 expression may also be used to monitor the treatment outcomes. It is known that LPS could induce inflammation to promote CHD [15] and regulate PVT1 expression [12]. We showed that LPS treatment decreased PVT1 expression in cardiomyocytes. Therefore, altered PVT1 expression in CHD is likely induced by LPS. However, our study is limited by the small sample size. In addition, the function of PVT1 in CHF-induced CKD and the molecular mechanism remain unclear. Our future studies will explore the possible functions and mechanisms.

## Conclusion

PVT1 is downregulated in CHF-induced CKD, and altered PVT1 expression has diagnostic and predictive values for the development of CKD in CHF patients.

## Data Availability

The data that support the findings of this study are not publicly available due to their containing information that could compromise the privacy of research participants, but are available on request from the corresponding author: Yaguang Guo, Department of Nephrology, Affiliated Hospital of Shaoxing College of Arts and Sciences, No.999 South Central Road, Shaoxing 312,000, Zhejiang Province, P. R. China. E-mail: yaguangguoscience@163.com.
